# Impairments in theory of mind following traumatic brain injury: A systematic review

**DOI:** 10.1192/j.eurpsy.2021.1959

**Published:** 2021-08-13

**Authors:** R. Chikramane

**Affiliations:** Consultation Liaison Psychiatry Department, Westmead hospital, Westmead, Australia

**Keywords:** theory of mind+ traumatic brain injury, theory of mind+ brain damage, theory of mind+ head injury

## Abstract

**Introduction:**

Theory of Mind (ToM) enables one to reflect upon the thoughts and emotions of others and oneself. Brain damage can lead to impaired social cognition resulting from ToM deficits. Studies examining ToM in patients with Traumatic Brain Injury (TBI) have yielded conflicting findings.

**Objectives:**

To assess the nature and extent of Theory of Mind (ToM) impairments post-TBI.

**Methods:**

Electronic databases searches included PubMed/MEDLINE, PubMed Central, Scopus, PsychArticles, PsychINFO, Web of Science, ProQuest Central, and Wiley Online Library databases. Only studies conducted on adult patients with TBI compared with healthy controls published in English in peer-reviewed journals were considered. Reference lists were manually checked for additional studies. 19 studies were identified.

**Results:**

Marked moderate-to-severe ToM deficits in adults post-TBI were observed across all severities of injury and chronicity. ToM deficits were documented across tasks and reflected a hierarchy where performance worsened significantly as tasks progressed in complexity. Despite supportive factors, certain aspects of ToM impairment, such as ability to detect and interpret non-literal speech and judge appropriateness of context remained affected in the subjects.

**Conclusions:**

ToM deficits represent a robust finding in adults with TBI. The chronicity of TBI requires a long-term view and is complicated by the fact that ToM deficits are invisible and difficult to understand. Perceptive-taking deficits faced by TBI sufferers has bio-socio-economic implications. This review also discusses implications for basic and clinical neuropsychology and rehabilitation efforts. Further research is needed, particularly in the form of large, longitudinal studies that mimic day-to-day interactions, to inform/support rehabilitation programs.

**Disclosure:**

No significant relationships.

